# Development of a Korean Hyperthyroidism-Specific Health Status Scale

**Published:** 2019-05

**Authors:** Soongyo YEOUM, Sunah PARK

**Affiliations:** 1. Department of Nursing, Seoil University, Seoul, South Korea; 2. Department of Nursing, Gangneung-Wonju National University, Wonju, South Korea

**Keywords:** Health status, Hyperthyroidism, Reliability, Scale, Validity

## Abstract

**Background:**

The aims of this study were to develop a hyperthyroidism-specific health status scale of Korea (K-HHSS) and to verify its validity and reliability.

**Methods:**

A methodological study was performed with hyperthyroid patients to assess the following properties: content validity, item analysis, Cronbach’s α, intraclass correlation coefficients, and confirmatory factor analysis. The data were obtained from 80 patients with hyperthyroidism given medical care at C university hospital in Seoul in 2017.

**Results:**

The construct validity was supported by the item-analysis correlations ranging between 0.31 and 0.82. The internal consistency reliability was from 0.70 to 0.85, and the scale’s stability was confirmed by intraclass correlation coefficients between 0.57 and 0.97. Construct reliability was 0.81 and the average variance extracted (AVE) was 0.72. These inclusion criteria resulted in the selection of 30 items in 7 categories.

**Conclusion:**

This scale will be useful as a limited health-measuring index for the nursing assessment of patients with hyperthyroidism in Korea.

## Introduction

Thyroid disorder, experienced by about 3% of Korean adults, is a more common chronic disease than cancer, which has a prevalence of 2.7% (1, 2). The prevalence of hyperthyroidism, one type of thyroid disorder, was 3.40 per 1,000 population in Korea (3) and about 20–93 Caucasians per 100,000 have had the disease, depending on the level of iodine intake (4, 5). Given that geographical region and race have no impact on the prevalence of hyperthyroidism, the prevalence of this condition in Korea would be similar (6).

Thyroid hormones have a broad impact on human organs (6, 7). Thus, the signs and symptoms of hyperthyroidism are also highly diverse. Impaired thyroid function can increase the risk of developing cardiovascular disease, osteoporosis, mental disorders, and disease of the central nervous system (8–12). Symptoms of hyperthyroidism often develop so insidiously that they go unnoticed and are frequently confused with other health problems. Thus, a sensitive health status scale to assess patients with hyperthyroidism could ameliorate the risk of complications, improve health status, and be immensely helpful in extending healthy life expectancy.

To date, no Korean scale has been developed to assess hyperthyroidism. The Hyperthyroidism Complaint Questionnaire (13) is used in other countries. It comprises only residual physical complaints and psychosocial problems after treatment for hyperthyroidism. Even though the results of a comparison with healthy adults has been published, whether internal consistency can be found in all items is still a matter of dispute. Graves’ Ophthalmopathy Quality of Life (14) is a disease-specific tool developed for patients with ophthalmopathy, who provide sixteen dichotomous answers to questions related to visual functioning and appearance. It was based on open-ended questionnaires only from 24 patients. However, its function is limited to measuring the quality-of-life associated with ocular disease, and it’s content validity is unclear. The Hyperthyroid Symptom Scale (15) is a very simple tool with only ten questions, particularly designed for those untreated Graves’ disease. This scale was sensitive to changes in both the adrenergic and metabolic components of hyperthyroidism, but not related to thyroid hormone levels in the initial assessment of disease.

The existing tools are not yet sufficient to support validity and reliability measures, such as content validity, internal consistency, and problems associated with the number of items. Most hyperthyroidism assessment tools have been developed in Western countries consisting, mainly on a Caucasian sample. In addition, among the classical symptoms of hyperthyroidism, hyperactivity is rarely observed in Asians (16–18), while ophthalmopathy occurs more frequently and more severely in Caucasians (6, 14). Considering that, 94% of the Korean hyperthyroidism is due to Graves’ disease, which causes ophthalmopathy (6), it is necessary to develop tools whose validity and reliability have been tested more completely and that are based on extensive patient reports and include each body system without negligence. The health status perceived by those with hyperthyroidism covers health problems from physical, social, and emotional aspects more extensively, in addition to the existence of the disease or symptoms. Therefore, the health status of patients may be more valuable than the evaluation of the results of biochemical tests.

Thus, the purpose of this study was to develop a scale to assess the health status of Korean patients with hyperthyroidism and to determine the validity and reliability of the scale. This scale encompasses the extensive impacts of thyroid hormone on peripheral tissue categorized according to body area. Therefore, this scale is configured to assess care issues without negligence, as well as to identify the health status of subjects.

## Methods

This work is a methodological study to develop a Korean hyperthyroidism-specific health status scale (K-HHSS).

### Item generation

The researchers reviewed studies reporting the consequences of a hyperthyroid state, overall health status issues, and classical hyperthyroid symptoms, as well as existing questionnaires, in order to identify relevant issues for patients with hyperthyroidism (6–17, 19–21).

The contents of the questionnaire were developed based on the signs and symptoms and the physical, mental, and social health problems caused by the excessive secretion of thyroid hormone. After reconfirming the frequently observed contents reported in the literature, questions were categorized according to each body system

**Content validity:** To determine if the questions in the scale properly represent the attribute or concept being measured, the content validity was examined based on experts’ inter-subjective decisions. The content validity index (CVI) was estimated after asking two experts to grade the relationships with hyperthyroidism. Then, only questions with a CVI of 0.60 or higher were selected (22). The experts, also highly qualified endocrinologist, have been examining patients with thyroid disorder for 25–30 years at a university hospital in Seoul. The items were then more precisely revised and corrected according to a review of all the open-ended questions, based on empirical opinions from experts on questions with similar definitions and on unrelated and unusual questions. Based on this process, a self-reporting scale was developed with a 5-point Likert scale. A high score signifies poor health status.

### Validity

This scale was evaluated by asking the subjects to scrutinize their signs and symptoms of hyperthyroidism and their health status when they were being diagnosed, as well as those they experienced before they received to care for their hyperthyroidism. After being diagnosed with hyperthyroidism, the subjects had continuously controlled serum thyroid hormone concentrations, as measured by a radioimmunoassay, using thyrotropin(TSH) at 0.17 mlU/ml or less and T4 at 1.86 ng/dl and higher.

**Construct validity**: Given that signs, symptoms, and health status can vary even among patients with hyperthyroidism (17), item analysis was employed to verify construct validity, used to evaluate whether each item meets the original purposes of measuring or diagnosing possible issues. Using classical test theory, it aims to identify the items with sufficient internal consistency and exclude items that do not satisfy this criterion (item discrimination) (23). The correlation coefficient for individual items should be at least .30. Those items that failed to meet this criterion were excluded (22).

**Convergent validity:** Convergent validity was examined by confirmatory factor analysis. Only questions whose factor loading was .4 or higher, with a significance level less than .05, were selected.

### Reliability

**Internal consistency:** Cronbach’s α was chosen to test the reliability of individual items, using a cutoff of .60 (24).

**Test-retest reliability**: An intraclass correlation coefficient (ICC) reflects the degree of reliability as follows: excellent, >.75; fair to good, .40–.75; and poor, <.40 (25). The test-retest reliability was measured twice. The second instance was 2 wk later. Since having a large number of cases is not necessary for calculating ICCs (25), the scale was forwarded to 40 people, and 26 questionnaires were returned (response rate, 65%); of these, 24 completed questionnaires were used for the ICC.

### Data collection and statistical analysis

The data were obtained from 80 patients with hyperthyroidism given medical care at C university hospital in Seoul in 2017. The sample size calculation, using the G*Power 3.1.2 program, indicated that a minimum sample size of 78 was required for a significance level of 0.05, effect size of 0.35, and 90% power.

This research was conducted after the consideration of ethical problems. The subjects were informed of their right to refuse to participate in this research.

The data collection was conducted in an outpatient waiting room over a 10–15-min period by experienced research staff. Whether individuals were appropriate, as research subjects were determined only after the researchers received their blood test results at the time of patients’ diagnosis. Before collecting data, prior consent to participate was received from the subjects.

The collected data were analyzed using SPSS-WIN (20.0) and AMOS (20.0). The CVI was estimated based on the ratios for the items that the experts considered to be are relevant to hyperthyroidism. In terms of a construct validity test, the correlation coefficient was analyzed based on corrected item-total correlation using item analysis. Cronbach’s α was used as a reliability test and ICC for test-retest reliability. Confirmatory factor analysis was used to examine convergent validity.

## Results

### Demographics

The subjects comprised 64 women (80.0%) and 16 men (20.0%). The greatest proportion of the subjects were in their 50s (31.3%) and based on body mass index, most were classified as normal (47.5%). The most common duration since the onset of hyperthyroidism was at least 5 yr (56.3%). The majority of the subjects had normal TSH levels (45.0%) and normal free-T4 levels (87.5%) (Table 1).

**Table 1:** Demographics of subjects (N = 80)

***Classification***	***n (%)***
Gender	
Male	16 (20.0)
Female	64 (80.0)
Age (yr)	
< 30	8 (10.0)
30–< 40	12 (15.0)
40–< 50	21 (26.2)
50–< 60	25 (31.3)
≥ 60	14 (17.5)
Latest BMI (kg/m^2^)	
< 18.5	7 (8.7)
18.5– < 23	38 (47.5)
23– < 25	11 (13.8)
≥ 25	13 (16.2)
**Untested**	11(13.8)
Period of disease (yr)	
< 1	5 (6.2)
1– < 2	9 (11.3)
2– < 5	21 (26.2)
≥ 5	45 (56.3)
Latest TSH (mlU/ml)	
< 0.17	11 (13.8)
0.17– < 4.05	36 (45.0)
≥ 4.05	7 (8.7)
**Untested**	26 (32.5)
Latest free-T4 (ng/dl)	
< 0.85	2 (2.5)
0.85– < 1.86	70 (87.5)
≥ 1.86	7 (8.7)
**Untested**	1(1.3)

The 51 questions in 8 categories were constructed for the complete scale. The items were finalized after revising the content to eliminate jargon and ambiguity.

### Content validity

Questions 38 (muscle tension) and 51 (hallucination) yielded CVI scores of .60 or less, and hence, these were eliminated (Table 2).

**Table 2:** Item analysis of the Korean hyperthyroidism-specific health status scale (K-HHSS) (N = 80)

***Items***	***Corrected item-total correlation***
18. I feel like my eyeballs will pop out.	0.82
4. I lack a familiar sense of self and attitude.	0.76
32. My breasts are enlarging (male).	0.74
19. I feel like my eyelids are swollen.	0.73
46. I am agitated often.	0.71
11. I have vitiligo around my hands or feet.	0.64
23. I find it difficult to breath during exercise.	0.64
20. I feel like my eyelids are sagging.	0.64
14. My nails get dry and cracked.	0.63
47. I lose concentration.	0.63
8. My skin is warm.	0.62
17. My face gets flushed.	0.62
29. I have frequent and loose bowel movements (diarrhea).	0.60
25. My appetite is increased.	0.60
49. I talk faster and faster.	0.59
50. I suffer from insomnia.	0.59
2. I have limitations in my usual activities.	0.56
41. I engage in over-activity.	0.55
48. I get confused.	0.55
40. I have hand tremors.	0.55
42. I am oversensitive.	0.54
27. I often get thirsty.	0.53
7. I have increased sweating.	0.52
39. My legs and arms get numb when going to bed and getting up in the morning.	0.51
13. My hair is falling out.	0.51
35. I find it difficult to sit and stand due to weak muscles, especially in the legs.	0.50
9. My skin is soft and tender.	0.50
1. I think my health has generally deteriorated.	0.49
33. My sexual desire has decreased.	0.45
43. I get excited often.	0.45
15. My nails grow fast.	0.44
44. I feel good.	0.43
34. I feel tired.	0.41
36. I find it hard to go up stairs due to weak muscles, especially in the legs.	0.40
22. I have photophobia.	0.39
6. I find the heat unbearable.	0.37
21. My eyes are bloodshot.	0.36
30. I have to urinate frequently.	0.35
24. I feel my heart beating in my chest.	0.35
28. I drink a lot of water.	0.31

### Construct validity

Item analysis was evaluated based on the correlation coefficients measured for corrected item-total correlations, which ranged from .04 to .82. 9 items had an *r* value of .30 or below and then, deleted the following items: questions 3, 12, 5, 37, 10, 31, 16, 45, and 26 (Table 2).

### Internal consistency

Three questions whose corrected item-total correlations were 0.30 or less (questions 11, 30, and 44) were eliminated. Cronbach’s α for the genitourinary system was .60 or less and thus, two additional items were removed (questions 32 and 33). This entire category was removed (Table 3).

**Table 3:** Internal consistency and test-retest reliability of the K-HHSS

***Domain***	***Items***	***M ± SD***	***Corrected item-total correlation***	***Cronbach’s α***	***ICC***	***95% CI***
GA	1. I think my health has generally deteriorated.	3.13 ± 1.34	.68		.68	.24–.86
	2. I have limitations in my usual activities.	2.49 ± 1.33	.66		.57	.01–.82
	4. I lack a familiar sense of self and attitude.	2.40 ± 1.26	.68	.80	.61	.80–.83
	6. I find the heat unbearable.	3.39 ± 1.45	.50		.94	.85–.97
SA	7. I have increased sweating.	3.37 ± 1.39	.42		.92	.82–.97
	8. My skin is warm.	3.05 ± 1.39	.54		.66	.19–.85
	9. My skin is soft and tender.	2.30 ± 1.16	.41		.86	.67–.94
	13. My hair is falling out.	3.05 ± 1.48	.45	.76	.77	.44–.90
	14. My nails get dry and cracked.	2.22 ± 1.37	.49		.88	.72–.95
	15. My nails grow fast.	1.97 ± 1.04	.53		.88	.71–.95
	17. My face gets flushed.	2.89 ± 1.40	.50		.87	.70–.95
OP	18. I feel like my eyeballs will pop out.	2.31 ± 1.46	.67		.97	.92–.99
	19. I feel like my eyelids are swollen.	2.48 ± 1.42	.63		.88	.71–.95
	20. I feel like my eyelids are sagging.	2.21 ± 1.29	.54	.83	.61	.07–.83
	21. My eyes are bloodshot.	2.65 ± 1.44	.70		.93	.83–.97
	22. I have photophobia.	2.65 ± 1.41	.63		.85	.65–.94
RC	23. I find it difficult to breath during exercise.	2.79 ± 1.38	.55		.91	.78–.96
	24. I feel my heart beating in my chest.	3.51 ± 1.26	.55	.70	.96	.91–.98
GI	25. My appetite is increased.	2.91 ± 1.36	.35		.86	.69–.94
	27. I often get thirsty.	2.87 ± 1.33	.55		.90	.77–.96
	28. I drink a lot of water.	3.03 ± 1.48	.48	.70	.87	.70–.95
	29. I have frequent and loose bowel movements (diarrhea).	2.33 ± 1.39	.42		.95	.89–.98
NM	34. I feel tired.	4.08 ± 1.04	.53		.92	.80–.97
	35. I find it difficult to sit and stand due to weak muscles, especially in the legs.	2.61 ± 1.47	.57		.82	.57–.92
	36. I find it hard to go up stairs due to weak muscles, especially in the legs.	2.86 ± 1.48	.50		.88	.71–.95
	39. My legs and arms get numb when going to bed and getting up in the morning.	1.73 ± 1.06	.44		.71	.32–.88
	40. I have hand tremors.	2.63 ± 1.50	.47	.73	.94	.85–.97
	41. I engage in over–activity.	2.43 ± 1.24	.32		.90	.77–.96
PB	42. I am oversensitive.	3.46 ± 1.37	.65		.90	.75–.96
	43. I get excited often.	3.28 ± 1.36	.75		.90	.77–.96
	46. I am agitated often.	2.66 ± 1.25	.66		.74	.38–.89
	47. I lose concentration.	3.20 ± 1.30	.59	.85	.80	.53–.92
	48. I get confused.	2.29 ± 1.25	.58		.67	.22–.86
	49. I talk faster and faster.	2.42 ± 1.39	.56		.91	.79–.96
	50. I suffer from insomnia.	2.69 ± 1.50	.54		.94	.86–.97

N = 80 //

a GA, general-aspects; GI, gastrointestinal system; NM, neuromuscular system; OP, ophthalmopathy; PB, psycho-emotional problems; RC, respiratory/ cardiovascular system; SA, skin/ appendages //

b The eliminated items due to their inter-item correlation coefficients ≤ .30: questions 11, 30, 44 //

c The removed items due to Cronbach’s α ≤ .60: questions 32, 33

### Test-retest reliability

The ICC values ranged from .57 to .97. Of the 35 questions, 78% (27 items) had excellent reliability, while 22% (8 items) demonstrated fair to good reliability (Table 3).

### Convergent validity

Confirmatory factor analysis was performed to validate the structure of the 35-item-7-factor scale. Five questions (7, 27, 40, 41, and 42) whose factor loadings were below .4 were excluded.

**Fig. 1:**
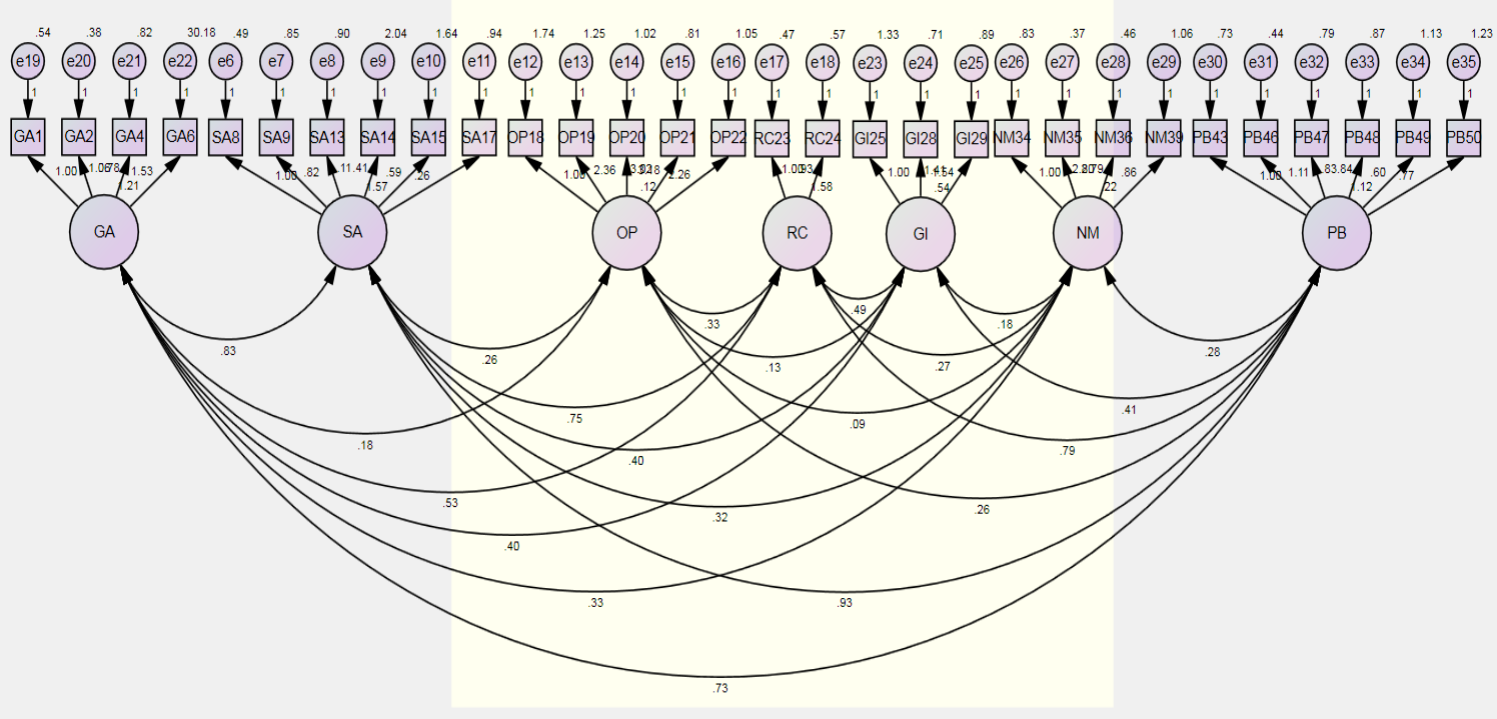
Confirmatory Factor Analysis for Convergent Validity

The total Criterion Ratio (CR) was significant, the average variance extracted (AVE) was .72, and construct reliability was .81 (*χ*^2^ = 715.949, *P*<.001, GFI=.93, AGFI=.92, PGFI=.92, CFI=.92, RMSEA=.08). The convergent validity of the scale was thus confirmed at the reference level or higher (Table 4, Fig. 1).

**Table 4:** Convergent validity of the K-HHSS

***Items***	***Estimates***	***Standard Error***	***Critical Ratio***	***P***	***Standardized Estimates***	***AVE***	***Construct Reliability***
GA 1	1.00				.83	.72	.81
GA 2	1.06	.12	8.54	< .001	.89		
GA 4	.78	.12	6.51	< .001	.69		
GA 6	1.53	.61	2.51	.012	.29		
SA 8	1.00				.87		
SA 9	.81	.12	6.72	< .001	.74		
SA 13	.30	.09	2.19	.035	.24		
SA 14	.42	14	2.88	.004	.34		
SA 15	.59	.14	4.31	< .001	.50		
SA 17	.26	.10	2.66	.008	.32		
OP 18	1.00				.25		
OP 19	2.36	1.18	1.99	.046	.59		
OP 20	3.02	1.47	2.05	.040	.72		
OP 21	3.18	1.54	2.07	.039	.77		
OP 22	2.26	1.13	2.00	.045	.60		
RC 23	1.00				.88		
RC 24	0.94	.12	7.62	< .001	.84		
GI 25	1.00				.54		
GI 28	1.41	.33	4.23	< .001	.78		
GI 29	1.55	.37	4.22	< .001	.77		
NM 34	1.00				.46		
NM 35	2.80	.67	4.19	< .001	.91		
NM 36	2.80	.67	4.18	< .001	.89		
NM 39	.86	.32	2.65	.008	.37		
PB 43	1.00				.78		
PB 46	1.11	.14	8.15	< .001	.87		
PB 47	.83	.13	6.38	< .001	.70		
PB 48	.84	.13	6.25	< .001	.69		
PB 49	.60	.13	4.47	< .001	.51		
PB 50	.77	.15	5.27	< .001	.59		
*χ*^2^ = 715.95, P<.001, GFI = .93, AGFI = .92, PGFI = .92, CFI = .92, RMSEA = .08							

Total CR ≥ 1.965, P<.05

## Discussion

The K-HHSS developed in this paper is a self-report questionnaire, with 30 items in 7 categories scored using a 5-point Likert-type scale. Based on previous studies, existing tools, and expert’s opinions (6–10, 12–14, 17, 19–21, 26), seven factors were constructed.

The classical symptoms of hyperthyroidism include heat intolerance, increased sweating, increased appetite, diarrhea, hand tremors, hyperactivity, and palpitations based on the classification by Watt et al. (17), which examined 75 references from 2,033 studies related to thyroid disorders.

The problems in the general-aspect category experienced by our subjects, other than the widely known classical symptoms, have also been found in other studies to “reduce general health perception,” “limit usual activities,” and “reduce the familiar sense of self‘,” and can usually be applied to those with ophthalmopathy (19, 21). In the skin/ appendages category, “skin warmth,” “soft and smooth feeling,” “flushness,” “fast nail growth,” and “change in nail texture” are the most frequently occurring symptoms in up to 84% of the subjects (17). Because of the sudden change in thyroid metabolism, even though it is temporary, “hair loss” has also been commonly reported by up to 31% of subjects (6). In the ophthalmopathy category, symptoms such as exophthalmos could occur before or after other symptoms of hyperthyroidism (6, 21). In the respiratory/cardiovascular systems category, “difficulty breathing during exercise” has also been indicated as a common symptom in other studies. This item demonstrated high sensitivity (16, 26). In the gastrointestinal system category, “polydipsia” displayed high discriminant power with 79% sensitivity (6). In the neuromuscular system category, “fatigue” from “weak muscles” is often felt in the lower extremities and causes discomfort in walking and daily routines. Overall, 53% of the subjects insisted that their lack of energy made them unable to perform work that they would normally do otherwise (13). Approximately 90% of inpatients experienced flaccid paralysis like “numbness” while asleep or at dawn, but this phenomenon was rarely observed while moving (6). There is a wide variation in the psycho-emotional problems category experienced. Similar studies have frequently found emotional lability (45%–99%) and anxiety/nervousness (30–100%) (17). These symptoms remained in 36%–46% and 25%–41% of the subjects, respectively (13), even after medical care, causing discomfort. Classical symptoms such as problems with “increased sweating,” “hyperactivity,” and “hand tremors” were not observed in our subjects. Studies on sweating issues have produced divergent results. Some studies have indicated frequent symptoms in all age groups and others indicated that these symptoms rarely occurred in the elderly (16, 18). Therefore, sweating issue should be understood as an age-related symptom. “Hand tremors” often occur when patients extend their arms due to hyperreflexia. Considering an occurrence of tremors in 58% of Panita et al.’s subjects (16), this item should be reclassified with the condition of extending the arms. “Hyperactivity” was an item rarely experienced by Asians (0.5% prevalence), although typical in Caucasian subjects (16–18). It is thus necessary to consider ruling out this item from the classical symptoms for Korean subjects based on the current validity and reliability.

Hyperthyroidism associated health status may differ between ethnicities (16). Therefore, it is necessary to identify continuously common issues for an ethnic group. This study is limited by the lack of a control group from a healthy population. Known group validity was also not evaluated since there was no scale to screen the overall health status of patients with hyperthyroidism. We are considered more reasonable to identify convergent validity than discriminant validity, for the correlation between the factors by itself will be higher in same type of disease.

The sign and symptoms of hyperthyroidism often changed from one hormone status to another due to treatment results. Therefore, thyroid status should be screened using a tool that covers the impacts on different body systems before, after, and during treatment. It will be a useful adjunctive clinical tool to assess patients with hyperthyroidism and the response to various types of therapy. This tool can be used to identify age-associated health status through research with the youth and elderly.

## Conclusion

The K-HHSS is useful as a limited-health measuring index, along with biochemical results, for the nursing assessment of Korean patients with hyperthyroidism. In addition, a score that measures the subject’s health status is beneficial for minimizing discrepancies between caregivers. This patient-reported scale, categorized according to body area, can simultaneously promote nursing assessment and nursing interventions.

## Ethical considerations

Ethical issues (Including plagiarism, informed consent, misconduct, data fabrication and/or falsification, double publication and/or submission, redundancy, etc.) have been completely observed by the authors.

## References

[1] National Cancer Informational Center (2015). [cited 24 Dec. 2015.] https://www.cancer.gov/aboutcancer/understanding/statistics

[2] National Health Insurance Service (2015). [cited 12 Oct. 2015]. https://www.nhis.or.kr/menu/boardRetriveMenuSet.xx?menuId=F3321

[3] Seo GH Kim SW Chung JH (2013). Incidence & prevalence of hyperthyroidism and preference for therapeutic modalities in Korea. J Korean Thyroid Assoc, 6(1):56–63.

[4] Bulow PI Knudsen N Jorgensen T (2002). Large differences in incidences of overt hyper-and hypothyroidism associated with a small difference in iodine intake: a prospective comparative register-based population survey. J Clin Endocrinol Metab, 87(10): 4462–4469. 12364419 10.1210/jc.2002-020750

[5] Empson M Flood VM Eastman CJ Mitchell P (2007). Prevalence of thyroid disease in an older Australian population. Intern Med J, 37(7): 448–455. 17547723 10.1111/j.1445-5994.2007.01367.x

[6] Jo B. H Clinical Thyroidology. Seoul, Korea: Medical Book; 2005.

[7] Iglesias P Diez JJ (2009). Thyroid dysfunction and kidney disease. Eur J Endocrinol, 160(4): 503–515. 19095779 10.1530/EJE-08-0837

[8] Siu CW Yeung CY Lau CP (2007). Incidence, clinical characteristics and outcome of congestive heart failure as the initial presentation in patients with primary hyperthyroidism. Heart, 93(4): 483–487. 17005710 10.1136/hrt.2006.100628 PMC1861478

[9] Boelaert K Franklyn JA (2005). Thyroid hormone in health and disease. J Endocrinol, 187(1): 1–15. 16214936 10.1677/joe.1.06131

[10] Demet MM Ozemen B Deveci A (2002). Depression and anxiety in hyperthyroidism. Arch Med Res, 33(6): 552–556. 12505101 10.1016/s0188-4409(02)00410-1

[11] Numbenjapon N Costin G Gilsanz V Pitukeheewanont P (2007). Low cortical bone density measures by computed tomography in children and adolescents with untreated hyperthyroidism. J Pediatr, 150(5): 517–530. 10.1016/j.jpeds.2007.01.045 17452230

[12] Tan ZS Beiser A Vasan RS (2008). Thyroid function and the risk of Alzheimer disease: The Framingham study. Arch Intern Med, 168(14): 1514–1520. 18663163 10.1001/archinte.168.14.1514 PMC2694610

[13] Fahrenfort JJ Wilterdink AM Van der Veen EA (2000). Long-term residual complaints and psychosocial sequelae after remission of hyperthyroidism. Psychoneuroendocrinology, 25(2): 201–211. 10674283 10.1016/s0306-4530(99)00050-5

[14] Terwee CB Gerding MN Dekker FW (1998). Development of a disease-specific quality of life questionnaire for patients with Graves’ ophthalmopathy: the GO-QOL. Br J Ophthalmol, 82(7): 773–779. 9924370 10.1136/bjo.82.7.773 PMC1722683

[15] Klein I Trzepacz PT Roberts M Levey GS (1988). Symptom rating scale for assessing hyperthyroidism. Arch Intern Med, 148(2): 387–390. 3124776

[16] Panita L Kittisak S Ajanee M Chaiyasit W (2006). Clinical manifestations of primary hyperthyroidism in the elderly patients at the out-patient clinic of Srinagarind hospital. J Med Assoc Thai, 89(2): 178–181. 16579003

[17] Watt T Groenvold M Rasmussen AK (2006). Quality of life in patients with benign thyroid disorders. Eur J Endocrinol, 154(4): 501–510. 16556711 10.1530/eje.1.02124

[18] Kim JH (2012). Thyroid dysfunction. J Korean Thyroid Assoc, 5(2): 94–98.

[19] Bianchi GP Zaccheroni V Solaroli E (2004). Health-related quality of life in patients with thyroid disorders. Qual Life Res, 13(4): 45–54. 15058786 10.1023/B:QURE.0000015315.35184.66

[20] Irwin K Kaie O (2001). Thyroid hormone and the cardiovascular system. N Engl J Med, 344(7): 501–509. 11172193 10.1056/NEJM200102153440707

[21] Park JJ Sullivan TJ Mortimer RH (2004). Assessing quality of life in Australian patients with Graves’ ophthalmopathy. Br J Ophthalmol, 88(1): 75–78. 14693779 10.1136/bjo.88.1.75 PMC1771927

[22] Lee UO Im NY Park HA (1998). Nursing, public health research and statistical analysis. Soomoonsa Seoul.

[23] Nunnally JC Bernstein IH (1994). Psychometric Theory. 3rd ed:. McGraw Hill New York.

[24] Hur MH Yang KS (2001). Multivariate data analysis. SPSS Academy Pub Co Seoul

[25] Han SY Nam JM Myoung SM Song KJ (2010). A Comparison of sample size requirements for intraclass correlation coefficient (ICC). KJAS, 23: 497–510.

[26] Osman F Franklyn JA Holder RL (2007). Cardiovascular manifestations of hyperthyroidism before and after anti-thyroid therapy: a matched case-control study. J Am Coll Cardiol, 49(1): 71–81. 17207725 10.1016/j.jacc.2006.08.042

